# Comparison of manual and semi-automated analysis tools for quantifying left ventricular volumes and mass in Children

**DOI:** 10.1186/1532-429X-13-S1-P358

**Published:** 2011-02-02

**Authors:** Jennifer A Bryant, Keith M Godfrey, Mark A Hanson, Sheila Barton, Charles R Peebles

**Affiliations:** 1 Southampton NIHR Nutrition, Diet & Lifestyle Biomedical Research Unit, Southampton, UK; 2 MRC Lifecourse Epidemiology Unit, Southampton, UK; 3 Southampton University Hospitals NHS Trust, Southampton, UK

## Introduction

CMR is an accurate and reproducible technique for the analysis of left ventricular volumes in adults but less well validated in children. Various analysis tools and segmentation methods are available but it is not clear which is most appropriate for use in children. Conventional manual segmentation tools require time-consuming contour tracing but allow some compensation for image mis-registration. The semi-automated tool (Argus 4D, Siemens Healthcare) employs a heart model based algorithm with reported significantly reduced analysis times but correction for the image mis-registration more frequently seen in children is more difficult.

## Purpose

To compare manual and semi-automated analysis tools used for the assessment of left ventricular (LV) volumes and mass measured using cardiovascular magnetic resonance (CMR).

## Methods

CMR was performed in 10 healthy children aged 9 years as part of a study of developmental influences on cardiovascular structure and function. Contiguous short axis steady state free precession LV cine images were acquired. Scans were repeated following a short interval. Data sets were analyzed to calculate LV volumes and mass using a manual technique (Osirix) and a semi-automated technique (Argus 4D). Papillary muscles and trabeculae were included in the blood pool. LV stroke volumes (SV) from each technique were compared with aortic flow data derived from aortic valve phase contrast velocity flow mapping sequences (analyzed with Argus).

## Results

Using the Bland Altman method, the mean difference in SVs for Osirix and Argus 4D was 7ml, with Argus 4D generally measuring larger values. The estimated coefficient of variation for SV measurements calculated using Osirix was lower than that using Argus 4D (10.6% vs 13.1%). Mean differences between the Osirix and Argus 4D SVs and aortic flow were 1.4ml and 5.7ml, respectively.

## Conclusions

In children SV measurements tend to be more reproducible using an Osirix manual technique compared with a semi-automated tool (Argus 4D). Osirix derived SVs were more accurate than Argus 4D when compared with aortic phase contrast derived flow data.

